# Disseminated Mycobacterium bovis Infection: A Rare Complication of Bacillus Calmette-Guérin Immunotherapy

**DOI:** 10.7759/cureus.50407

**Published:** 2023-12-12

**Authors:** Marina Henriques Mendes, André Guimarães, Catrine Dahlstedt Ferreira, Fátima Carneiro, Lurdes Santos

**Affiliations:** 1 Internal Medicine, Centro Hospitalar Tâmega e Sousa, Penafiel, PRT; 2 Infectious Diseases, Centro Hospitalar de São João, Porto, PRT; 3 Pathology, Hospital Garcia de Orta, Lisbon, PRT; 4 Pathology, Centro Hospitalar de São João, Porto, PRT

**Keywords:** bacillus calmette-guérin immunotherapy, disseminated mycobacterium bovis infection, pulmonary micronodulation, hepatic nodules, granulomatous hepatitis

## Abstract

Intravesical instillation of bacillus Calmette-Guérin (BCG) is a crucial adjunctive therapy in the treatment of bladder carcinoma. Its systemic complications are rare but include disseminated *Mycobacterium* *bovis* (*M. bovis*) infections, the diagnosis of which is a challenging task that requires keen clinical awareness. We report the case of an adult male treated with BCG who presented with fever, nonspecific constitutional symptoms, hepatic cytolysis, and cholestasis. After a detailed workup, the diagnosis was made of disseminated *M. bovis* infection with hepatic, pulmonary, renal, and ureteral involvement. Prompt anti-tuberculosis treatment resulted in clinical and analytical improvement. This case highlights the importance of early recognition of this serious complication in patients with BCG exposure, as well as the difficulty in confirming the diagnosis for proper treatment.

## Introduction

Intravesical instillation of bacillus Calmette-Guérin (BCG), a live attenuated strain of *Mycobacterium bovis* (*M. bovis*), is the recommended immunotherapy for all high-grade and some low-grade non-muscle-invasive bladder cancer (NMIBC) [1]. In patients who receive maintenance BCG after transurethral resection, it markedly lowers the likelihood of recurrence and substantially reduces the risk of progression to muscle-invasive disease [2,3]. However, the high incidence of complications has been considered a primary factor contributing to low adherence to BCG therapy. Only 16% of patients undergoing maintenance BCG have managed to complete the full course of the three-year immunotherapy, primarily due to adverse events, as reported [4].

BCG-induced complications may manifest with a spectrum of symptoms, ranging from self-limited irritative voiding symptoms to potentially severe sepsis [5]. Less commonly observed (3-7%) systemic complications tend to be more severe and include disseminated *M. bovis* infection, persistent fever, and any organ involvement beyond the genitourinary system [6]. Identifying disseminated infections poses a diagnostic challenge since microbial evidence is often lacking. Thus, an accurate diagnosis necessitates heightened clinical suspicion regarding the potential involvement of BCG in the development of the condition.

## Case presentation

A 77-year-old man diagnosed with high-grade bladder urothelial carcinoma (pT1) was submitted to transurethral resection followed by immunotherapy, initially with BCG instillation, weekly for six weeks, followed by monthly for two months. A few days after the first monthly instillation, he presented with fever, severe fatigue, nausea, and anorexia. Due to the persistence of symptoms associated with weight loss, after the second monthly instillation, he went to the emergency department. On admission, he was febrile (38.3°C), with no other findings upon physical examination. Blood tests were performed and revealed mild anemia (hemoglobin (Hb) 12.5g/dL) with a normal white blood cell and platelet count, acute kidney injury (creatinine (Cr) 1.47mg/dL), increased levels of C-reactive protein (47.7mg/L), hepatic cytolysis, and cholestasis (AST 352 U/L, ALT 320 U/L, GGT 181 U/L, ALP 162 U/L) without hyperbilirubinemia. He was then admitted to the Infectious Diseases Department for further investigation.

**Figure 1:**
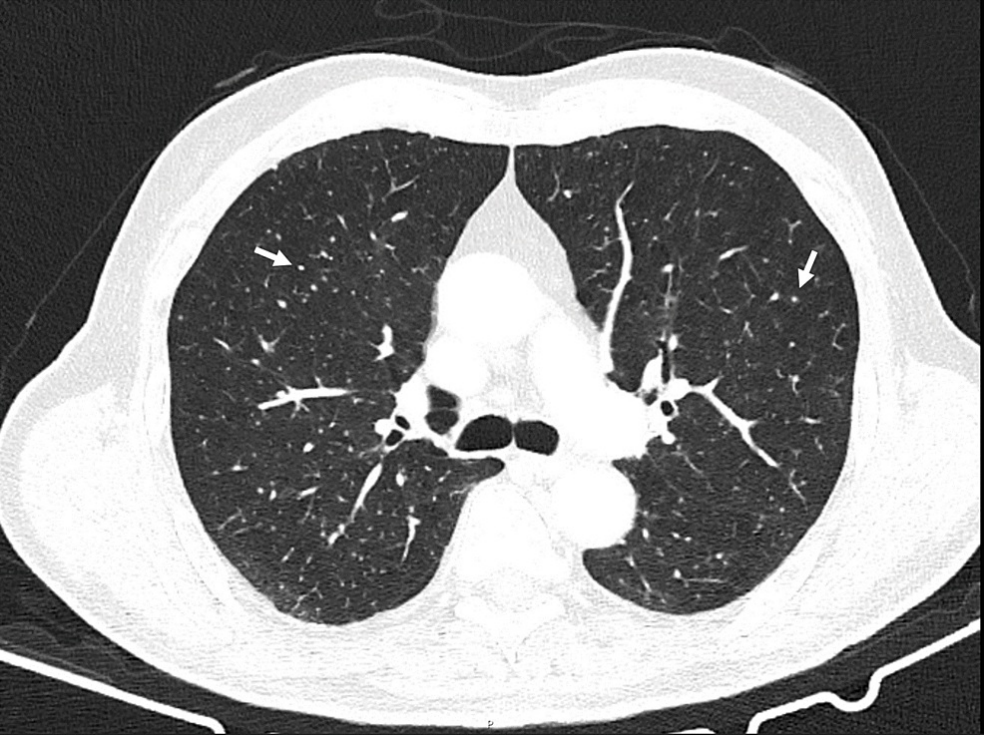
Thoracic CT scan showing pulmonary micronodulation (arrows) in a miliary pattern

**Figure 2:**
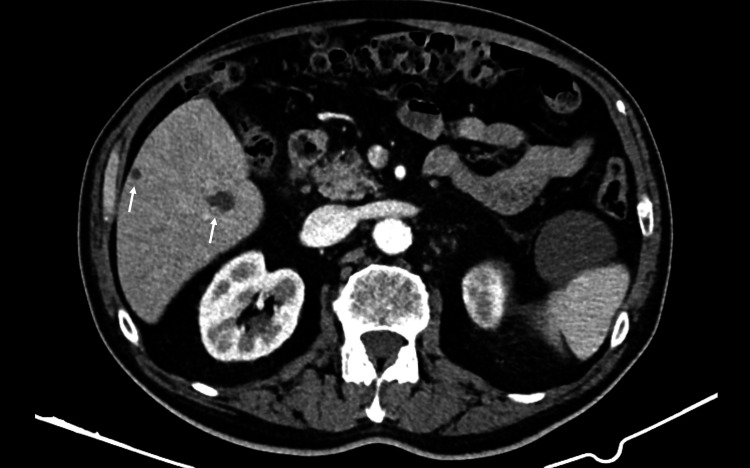
Abdominal CT scan showing hepatic nodules (arrows)

**Figure 3:**
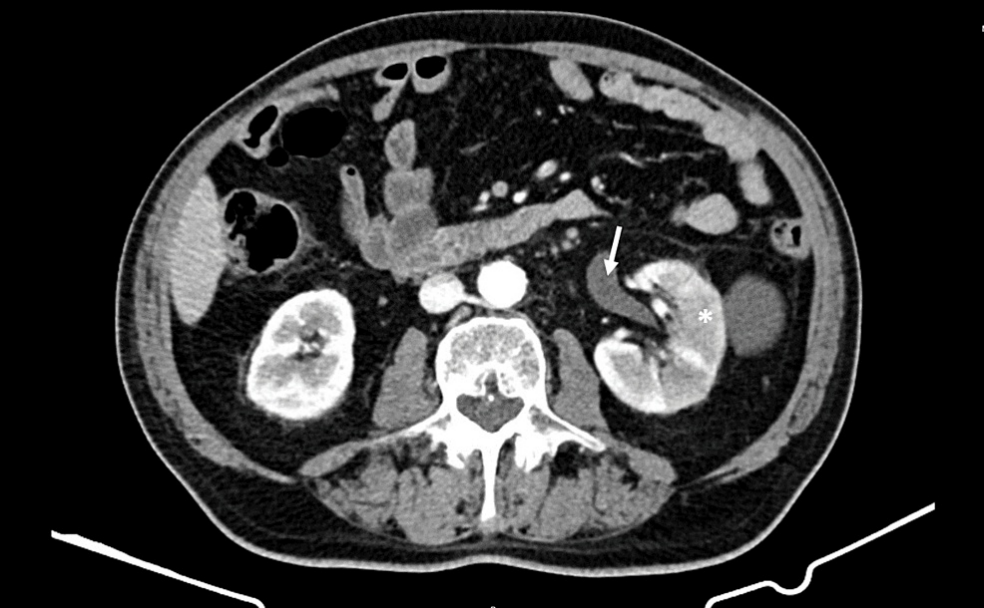
Abdominal CT scan showing areas of contrast attenuation in renal parenchyma (asterisk) and hydronephrosis of the left kidney (arrow)

**Figure 4:**
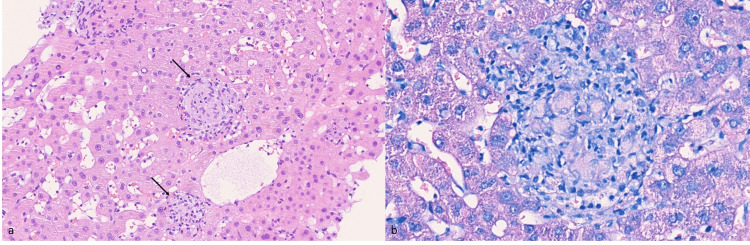
Liver biopsy (a) H&E stain of an adequate specimen, composed of liver parenchyma with multiple nonnecrotizing granulomas with Langhans cells (arrows); (b) Ziehl-Neelsen histochemistry was performed with a negative result

The thoracic and abdominal CT scans showed diffuse pulmonary micronodulation in a miliary pattern (Figure 1), several hepatic nodules (Figure 2), and areas of contrast attenuation in renal parenchyma and hydronephrosis of the left kidney with ipsilateral ureteral thickening (Figure 3). A nucleic acid amplification test in urine and sputum was positive for *M. bovis*. A hepatic biopsy was also performed. Histology revealed granulomatous hepatitis with giant Langhans cells (Figure 4), although with a negative Ziehl-Neelsen stain. The nucleic acid amplification test for the M. tuberculosis complex was negative in the liver biopsy. The diagnosis of disseminated *M. bovis* infection was made, and anti-tuberculosis therapy was instituted with clinical improvement and hepatic enzyme progressive normalization.

## Discussion

Intravesical BCG immunotherapy has demonstrated efficacy in preventing the recurrence and progression of NMIBC. Nevertheless, its utilization remains challenging due to the potential onset of a diverse range of complications that can manifest within days to years after the initiation of BCG instillation. Since the exact immune mechanism of BCG therapy remains elusive, the complete explanation of the pathogenesis of adverse reactions following intravesical BCG instillation is not yet fully understood. A debate persists between the inflammatory hypersensitivity hypothesis, backed by histological evidence of granulomas in the absence of microorganisms, and the bacterial invasion hypothesis, supported by reports of active bacilli found in various tissues [6].

Although serious complications are uncommon, every clinician faced with a patient with a history of intravesical BCG therapy should be aware of potential adverse events and management. Considering the fact that BCG infections and reactions can occur in any organ, a detailed medical history and further investigation provide critical clinical value. Despite the presence of obvious alarm symptoms attributed to BCG, confirming suspected complications related to BCG therapy may pose a challenge. Acid-fast bacilli staining, mycobacterial culture, and nucleic acid amplification testing are often negative. Tissue biopsies are often required to assess for non-caseating granuloma formation, and specimen cultures are useful to identify the presence of *M. bovis*.

The overall rate of positive findings is 48% in microbiological studies (acid-fast bacilli stain, culture, and nucleic acid amplification testing) and 65.5% in tissue biopsy, while the identification of granulomatous inflammation in specimens is available in 86.3% of cases [6]. While most side effects are typically self-limited, initiating anti-tuberculosis therapy promptly upon diagnosis is crucial to preventing the development of more severe complications. An essential consideration is that the prognosis of a complication often hinges on the prompt initiation of treatment. Therefore, maintaining a high level of clinical suspicion is crucial to preventing delays in management.

## Conclusions

This case highlights the importance of recognizing a disseminated *M. bovis* infection and the difficulty in diagnosing it, given its nonspecific clinical manifestations and the potential involvement of multiple organs. Nevertheless, the possibility of this diagnosis must not be overlooked in a patient undergoing BCG treatment who exhibits systemic inflammation and shows clinical or analytical signs of organ dysfunction. The challenge stems from differentiating this condition from other inflammatory and infectious conditions. Achieving a diagnosis requires a comprehensive approach that involves obtaining biopsies from the affected organs and conducting culture tests to confirm the presence of an *M. bovis* infection. This approach allows for the prompt initiation of appropriate treatment while discontinuing intravesical instillation of BCG.
